# Propagation and blocking of bistable waves by variable diffusion

**DOI:** 10.1007/s00285-025-02260-7

**Published:** 2025-11-05

**Authors:** Keita Nakajima, Hirokazu Ninomiya

**Affiliations:** 1 Graduate School of Advanced Mathematical Sciences, Meiji University, 4-21-1 Nakano, Nakano-ku, Tokyo, 164-8525 Japan; 2 School of Interdisciplinary Mathematical Sciences, Meiji University, 4-21-1 Nakano, Nakano-ku, Tokyo, 164-8525 Japan

**Keywords:** Reaction–diffusion equations, Propagation, Variable diffusion, Singular limit problem, Transition probability, 35K57, 35Q92, 35K67, 53E10

## Abstract

Biological diffusion processes are often influenced by environmental factors. In this study, we investigate the effects of variable diffusion, which depend on the point between the departure and the arrival points, on the propagation of bistable waves. This process includes neutral, repulsive, and attractive transitions. Using singular limit analysis, we derive the equation for the interface between two stable states and examine the relationship between wave propagation and variable diffusion. In particular, when the transition probability depends on the environment at the dividing point between the departure and the arrival points, we derived an expression for the wave propagation speed that includes this dividing point ratio. More specifically, the threshold between wave propagation and conditional blocking in a one-dimensional space occurs when the transition probability is determined by a dividing point ratio of 3:1 between the departure and the arrival points. Furthermore, as an application of this concept, we consider the Aliev-Panfilov model to explore the mechanism for generating spiral patterns.

## Introduction

Propagation phenomena such as the spread of infectious diseases, habitat invasion by living organisms, and propagation of action potentials in the heart are often observed in the natural world. These phenomena are typically modeled using partial differential equations that include reaction and diffusion terms, which are known as reaction–diffusion systems. If the media are homogeneous, traveling wave solutions are frequently employed to describe these propagation phenomena by reaction-diffusion systems. These solutions represent waves that move with a constant speed without changing their profiles. The balance between the diffusion and reaction terms significantly influences the propagation speed. In a more realistic model, heterogeneous media are present, along with movement behaviors in biological systems influenced by stimuli from heterogeneous media, such as taxis and kinesis. For instance, organisms may change their turning rate (klinokinesis) or move in response to the direction of chemical signals (chemotaxis). Understanding these movement behaviors and incorporating them into reaction-diffusion systems allows for a more comprehensive analysis of how organisms interact with their environments and respond to different stimuli, leading to a deeper understanding of the underlying mechanisms driving propagation phenomena.

Okubo and Levin in Okubo and Levin (2001) introduced biodiffusion using the transition probabilities. More precisely, there are the following three types of variable diffusion:

1.1 $$\begin{aligned} u_t= & \nabla (D(x)\nabla u)+g(u),\hspace{18mm} \ t>0,\ \ x\in \mathbb {R}^N,\end{aligned}$$

1.2 $$\begin{aligned} u_t= & \Delta (D(x) u)+g(u),\hspace{21mm} \ t>0,\ \ x\in \mathbb {R}^N,\end{aligned}$$

1.3 $$\begin{aligned} u_t= & \nabla (D(x)\nabla u-u\nabla D(x))+g(u),\ \ t>0,\ \ x\in \mathbb {R}^N, \end{aligned}$$

where *N* denotes the spatial dimension, *D*(*x*) represents spatial heterogeneity, and *g* is a reaction term. Here, the subscript *t* denotes differentiation with respect to the time variable. We assume that *D*(*x*) is a positive smooth function and *g* is a smooth function specified later. The first equation (1.1) is derived when the transition probabilities are assumed to equally depend on the average between the departure and the arrival points. This equation is often used when the flux is consistently directed from higher concentrations to lower concentrations. It is also known as Fick’s law. When the transition probabilities depend only on the conditions at the point of departure, we obtain (1.2). Chapman derived (1.2) using the kinetic theory to explain the thermal diffusion phenomenon in Chapman (1928). A typical application of this type is the cross-diffusion system proposed by Shigesada et al. (1979). By contrast, when the transition probabilities depend only on the conditions at the point of arrival, (1.3) is obtained. The Keller-Segel model uses this type of diffusion (e.g. see Keller and Segel (1970)).

Singular limit analysis is often effective for clarifying the influence of heterogeneity of the media, (see also Fife and Peletier (1981); Yanagida (1982); Fusco and Hale (1985); Angenent et al. (1987)). By introducing a small parameter appropriately in the reaction-diffusion equation, transition layers of solutions emerge, and the governing equation for the position of these layers can be obtained through the singular limit problem. The dynamics of the interface appearing in the singular limit problem of the Allen-Cahn equation is described by the mean curvature flow (e.g. see Fife (1988); De Mottoni and Schatzman (1995)). Ei, Iida and Yanagida in Ei et al. (1997) studied the singular limit problem of (1.1) with $g(u):=f(u,\varepsilon )/\varepsilon ^2$, namely,

$$\begin{aligned} u_t= & \nabla (D(x)\nabla u)+\dfrac{1}{\varepsilon ^2}f(u,\varepsilon ),\ \ t>0,\ \ x\in \mathbb {R}^N \end{aligned}$$

as $\varepsilon \rightarrow +0$, where *f* is a bistable nonlinearity under certain assumptions. They showed that the interface between two stable states, denoted by $\Gamma (t)$, is governed by

$$\begin{aligned} V=\left( c-\left\langle \nu , \nabla \sqrt{D(y)}\right\rangle \right) \sqrt{D(y)}-(N-1)D(y)\kappa ,\quad y\in \Gamma (t), \end{aligned}$$

where *V*, $\kappa (y)$, and $\nu (y)$ are the speed of the interface $\Gamma (t)$ at $y\in \Gamma (t)$ in the outer normal direction, the mean curvature and the outer normal unit vector at $y\in \Gamma (t)$. Here *c* is a constant related to the speed of the one-dimensional traveling wave solution (see Ei et al. (1997) or Sect. 2 for the details). This result for (1.1) implies that the gradient of *D* plays an important role on the dynamics of propagating waves. More precisely, consider one-dimensional wave moving to the right. The wave can be blocked at one of the points satisfying

$$\begin{aligned} c=\dfrac{D_x(x)}{2\sqrt{D(x)}}. \end{aligned}$$

This blocking phenomenon may cause the system to exhibit more complex dynamics in multi-dimensional cases. Thus, investigating the relationship between various patterns and heterogeneity of media has become an interesting and important problem. This naturally leads to the following question: *What about the other equations* (1.2), *and* (1.3)?  However, these equations may exhibit different behavior. To address this question, in this study, we consider the singular limit problem of the scalar reaction-diffusion equation with variable diffusion, including (1.1)–(1.3), as one of the simplest models in heterogeneous media and discuss the dynamics of the singular limit problem.

Since the movement of biological species involves various microscopic factors, it is difficult to appropriately capture their behavior at a macroscopic level. However, understanding the dynamics might provide valuable insights into the appropriate spatial arrangement of diffusion coefficients.

## Main results

To study (1.1)–(1.3) in an integrated manner, we revisit the derivation of variable diffusion from transition probabilities. For simplicity, we consider a discretized one-dimensional space. Let $u^n_j$ be the population density at time *n* and at position $x_j$. The probability of transition from $x_j\in \mathbb {R}$ to $x_k\in \mathbb {R}$ is denoted by $T(x_j,x_k)$. Then, considering the inflow and outflow at position $x_j$ at time $n+1$, we obtain the following equation as in Okubo and Levin (2001):

2.1 $$\begin{aligned} u^{n+1}_{j}-u^n_{j}=&T(x_{j-1}, x_j)u_{{j-1}}^n+T(x_{j+1}, x_j)u_{{j+1}}^n\\&-\left( T(x_j, x_{j-1})+T(x_j, x_{j+1})\right) u_{j}^n.\ \quad \end{aligned}$$

As mentioned before, the types of diffusion equations (1.1)–(1.3) are determined by how the transition probability is related to the departure and the arrival points. By generalizing this idea, we consider a case in which the transition probability depends on the point dividing the departure and the arrival points in the ratio of $1-\alpha$ to $\alpha$ where $0\le \alpha \le 1$. Namely, the transition probability is given by

$$\begin{aligned} T(x_j,x_k) = {{\mathcal {D}}}(\alpha x_j + (1-\alpha ) x_k). \end{aligned}$$

Therefore, substituting it into (2.1) yields

$$\begin{aligned} u(x,t+\Delta t) - u(x,t)= &\, {{\mathcal {D}}}(x-\alpha \Delta x) u(x-\Delta x,t)+{{\mathcal {D}}}(x+\alpha \Delta x) u(x+\Delta x,t)\\ & \quad -({{\mathcal {D}}}(x+(1-\alpha ) \Delta x)+{{\mathcal {D}}}(x-(1-\alpha ) \Delta x)) u(x,t), \end{aligned}$$

where $t = n \Delta t,\ x_j = x,\ x_{j\pm 1}=x\pm \Delta x$. Dividing both sides by $\Delta t$ and taking the limit as $\Delta x$ and $\Delta t \rightarrow 0$, we get

2.2 $$\begin{aligned} u_t=D(x)u_{xx}+2{ \alpha }D'(x)u_x+(2\alpha -1) u D''(x). \end{aligned}$$

Here, we used $D(x)=\lim _{{ \Delta t \rightarrow 0}} \Delta x ^2 {{\mathcal {D}}}(x) / \Delta t$. For the *N*-dimensional case, we obtain the following diffusion equation:

2.3 $$\begin{aligned} u_t=D(x)\Delta u+2\alpha \langle \nabla D(x),\nabla u\rangle +(2\alpha -1)u\Delta D(x). \end{aligned}$$

This is a natural extension of (1.1)–(1.3) because the above equation can be rewritten as

$$\begin{aligned} u_t=\alpha \Delta (D(x)u)+(1-\alpha )\nabla (D(x)\nabla u-u\nabla D(x)). \end{aligned}$$

Thus, the case $\alpha =1/2$ (resp. $\alpha = 1$, $\alpha =0$) corresponds to the neutral transition (1.1) (resp. the repulsive transition (1.2), the attractive transition (1.3)). For simplicity of notation, we define

2.4 $$\begin{aligned} {{\mathcal {L}}}_{D,\alpha } u:= & D(x)\Delta u+2\alpha \langle \nabla D(x),\nabla u\rangle +(2\alpha -1)u\Delta D(x), \end{aligned}$$

In Wereide (1914), Wereide introduced

2.5 $$\begin{aligned} u_t= & \nabla \left( \sqrt{D(x)}\nabla (\sqrt{D(x)}u)\right) +g(u),\ \ t>0,\ \ x\in \mathbb {R}^N. \end{aligned}$$

In Alfaro et al. (2022); Hilhorst et al. (2024), (2.5) is generalized to

2.6 $$\begin{aligned} u_t= & \nabla \Big (D(x)^{1-q}\nabla (D(x)^{q}u)\Big )+g(u),\ \ t>0,\ \ x\in \mathbb {R}^N \end{aligned}$$

for $$q\in \mathbb {R}$$. Since

$$\begin{aligned} \nabla \Big (D(x)^{1-q}\nabla (D(x)^{q}u)\Big ) =D(x)\Delta u+(q+1)\langle \nabla D(x), \nabla u\rangle +qu\Delta D(x), \end{aligned}$$

the generalized diffusion term of (2.6) coincides with (2.4) with $q=2\alpha -1$.

First consider the stationary solutions of (2.3) with zero-flux boundary condition in bounded domain. By using the boundary condition, we have

$$\begin{aligned} \alpha \nabla (D(x)u)+(1-\alpha )(D(x)\nabla u-u\nabla D(x))=0 \end{aligned}$$

in the domain, this implies that

$$\begin{aligned} u(x)=\mathrm{const.\,}D(x)^{1-2\alpha }. \end{aligned}$$

This suggests that the repulsive transition tends to move toward areas of lower diffusion, while the attractive transition tends to move toward areas of higher diffusion. Furthermore, from the perspective of stationary analysis, $\alpha =1/2$ represents the threshold between two movement tendencies.

To clarify the propagation phenomenon by this generalized diffusivity, we consider the following equation with a small positive parameter $\varepsilon$:

2.7 $$\begin{aligned} u_t={{\mathcal {L}}}_{D,\alpha } u+\dfrac{1}{\varepsilon ^2}f(u,\varepsilon ),\ \ t>0,\ \ x\in \mathbb {R}^N, \end{aligned}$$

where *D* is a positive smooth function in $\mathbb {R}^N$ and $D(x)\ge D_*>0$ and $|\Delta D(x)|\le D^*<\infty$ for any $x\in \mathbb {R}^N$. We also assume that *f* is a smooth function with exactly three zeros 0, 1, $a(\varepsilon )\in (0,1)$, and *f* satisfies

2.8 $$\begin{aligned} \left\{ \begin{array}{l} f(u,\varepsilon )=f_0(u)+\varepsilon f_1(u,\varepsilon ),\quad \displaystyle \int \limits _0^1f_0(u)du=0,\quad \displaystyle \int \limits _0^1f_1(u,0)du>0,\\ \displaystyle f(0,\varepsilon )=f(1,\varepsilon )=f(a(\varepsilon ),0),\quad f'(0,\varepsilon )<0,\quad f'(1,\varepsilon )<0,\\ f(u,\varepsilon )<0\quad \hbox {for }0<x<a(\varepsilon ),\quad f(u,\varepsilon )>0\quad \hbox {for }a(\varepsilon )<x<1, \end{array}\right. \end{aligned}$$

where $$f_0$$ and $$f_1$$ are smooth functions. Note that $$f_0(0)$$$$=f_0(1)$$$$=f_1(0,\varepsilon )$$$$=f_1(1,\varepsilon )$$ = 0 by (2.8). A typical example is given by

2.9 $$\begin{aligned} f(u,\varepsilon )=u(1-u)\left( u-\dfrac{1}{2}+{ a_1}\varepsilon \right) =u(1-u)\left( u-\dfrac{1}{2}\right) +{ a_1}\varepsilon u(1-u) \end{aligned}$$

with a positive constant ${ a_1}$. The equation is often referred to as the Allen-Cahn-Nagumo equation.

Next, we formally derive the singular limit problem as in Ei et al. (1997, Sect. 3). We assume that the level surface

$$\begin{aligned} \Gamma ^\varepsilon (t):=\left\{ x\ \Big |\ u(x,t)=\dfrac{1}{2}\right\} \end{aligned}$$

is an $(N-1)$-dimensional smooth hypersurface that is the boundary of a bounded open set in $\mathbb {R}^N$ and is diffeomorphic to a reference manifold $\bar{\Gamma }$ for every $t\in (0,T)$. There exists $\xi ^*>0$ such that $(-\xi ^*,\xi ^*)\times \bar{\Gamma }$ is diffeomorphic to the neighborhood of $\Gamma ^\varepsilon (t)$ by the mapping

$$\begin{aligned} X^\varepsilon (t,\xi ,\sigma ):=y^\varepsilon +\xi \nu (y^\varepsilon ;\Gamma ^\varepsilon (t)), \end{aligned}$$

where $y^\varepsilon =y^\varepsilon (t,\sigma )\in \Gamma ^\varepsilon (t)$. Let $({ \Xi ^\varepsilon }(t,\cdot ),\Sigma ^\varepsilon (t,\cdot ))$ be the inverse of $X^\varepsilon (t,\cdot ,\cdot )$. Namely, $x=X^\varepsilon (t,{ \Xi ^\varepsilon }(t,x),\Sigma ^\varepsilon (t,x))$. Set

$$\begin{aligned} v(t,\mu ,\sigma ;\varepsilon )=u(X^\varepsilon (t,\varepsilon \mu ,\sigma ),t)=u(y^\varepsilon +\varepsilon \mu \,\nu (y^\varepsilon ;\Gamma ^\varepsilon (t)),t). \end{aligned}$$

We note that $u(x,t)=v(t,\Xi ^\varepsilon (t,x)/\varepsilon ,\Sigma ^\varepsilon (t,x);\varepsilon )$. As in Lemma 3.1 of Ei et al. (1997) (see also De Mottoni and Schatzman (1995)), *v* satisfies the following equations for $(t,\mu ,\sigma )\in [0,T]\times (-\xi ^*/\varepsilon ,\xi ^*/\varepsilon )\times \bar{\Gamma }$,

2.10 $$\begin{aligned} \left\{ \begin{array}{l}v_t=\dfrac{D^\varepsilon }{\varepsilon ^2} v_{\mu \mu }+\dfrac{D^\varepsilon \Delta \Xi ^\varepsilon }{\varepsilon } v_\mu +D^\varepsilon B^\varepsilon (t,\mu )v +\dfrac{2\alpha }{\varepsilon }\langle \nabla D^\varepsilon ,\nu \rangle v_\mu +2\alpha \langle \nabla D^\varepsilon ,\nabla \Sigma ^\varepsilon \rangle \cdot v_\Sigma \\ \hspace{9mm}+(2\alpha -1)v\Delta D^\varepsilon -\dfrac{\Xi ^\varepsilon _t}{\varepsilon }v_\mu -\Sigma ^\varepsilon _t\cdot v_\Sigma +\dfrac{1}{\varepsilon ^2}f(v,\varepsilon ),\\ v(t,0,\sigma ;\varepsilon )=\dfrac{1}{2}, \end{array}\right. \end{aligned}$$

where $D^\varepsilon :=D(X^\varepsilon (t,\varepsilon \mu ,\sigma ))$, $B^\varepsilon$ is an elliptic operator on $\bar{\Gamma }$ as in Ei et al. (1997), and $\langle \nabla D^\varepsilon ,\nabla \Sigma ^\varepsilon \rangle \cdot v_\Sigma ,\ \Sigma ^\varepsilon _t\cdot v_\Sigma$ denote the derivatives of *v* in the direction of the vector field $\langle \nabla D^\varepsilon ,\nabla \Sigma ^\varepsilon \rangle$, $\Sigma ^\varepsilon _t$, respectively.

By expanding $v=U^0+\varepsilon U^1+\varepsilon ^2U^2\cdots$, we obtain

2.11 $$\begin{aligned} D(y^0)U^0_{\mu \mu }+f(U^0,0)=0 \end{aligned}$$

as the highest order term $O(\varepsilon ^{-2})$ of (2.10) where we used $X^0(t,0,\sigma )=y^0$. When $D(y^0)$ is replaced with 1, we denote the solution to the above equation as $\Phi$. Namely,

2.12 $$\begin{aligned} \Phi ''+f(\Phi ,0)=0,\quad \Phi (-\infty )=1,\ \Phi (0)=\dfrac{1}{2},\ \Phi (\infty )=0, \ \Phi '<0. \end{aligned}$$

From (2.11) and using the definition of $\Phi$, we have

$$\begin{aligned} U^0(\mu )=\Phi \left( \dfrac{\mu }{\sqrt{D(y^0)}}\right) . \end{aligned}$$

Plugging

$$\begin{aligned} &  {D^\varepsilon } v_{\mu \mu }{\varepsilon ^{-2}}=D(y^0)U^0_{\mu \mu }\varepsilon ^{-2}+D(y^0)U^1_{\mu \mu }\varepsilon ^{-1}+\mu \,\langle \nu ,\nabla D(y^0)\rangle U^0_{\mu \mu }\varepsilon ^{-1}+O(1),\\ &  f(v,\varepsilon )\varepsilon ^{-2}=f_0(U^0)\varepsilon ^{-2}+f_0'(U^0)U_1\varepsilon ^{-1}+f_1(U^0,0)\varepsilon ^{-1}+O(1) \end{aligned}$$

into (2.10), from $O(\varepsilon ^{-1})$, we obtain

2.13 $$\begin{aligned} &  {D(y^0) U^1_{\mu \mu }+\mu \,\langle \nu ,\nabla D(y^0)\rangle U^0_{\mu \mu }+D(y^0)\Delta \Xi ^0 U^0_\mu }\nonumber \\ &  \quad +2\alpha \langle \nabla D(y^0),\nu \rangle U^0_\mu -\Xi ^0_tU^0_\mu +f_0'(U^0)U_1+f_1(U^0,0)=0. \end{aligned}$$

Differentiating (2.11) in $\mu$ yields

$$\begin{aligned} D(y^0)U_{\mu \mu \mu }^0+f_0'(U^0)U^0_{\mu }=0. \end{aligned}$$

By multiplying (2.13) by $U^0_{\mu }$, and integrating over $\mu \in \mathbb {R}$, we obtain

$$\begin{aligned} & {\langle \nu ,\nabla D(y^0)\rangle \int _{\mathbb {R}}\mu U^0_{\mu \mu }U^0_{\mu }d\mu +D(y^0)\Delta \Xi ^0 \int _{\mathbb {R}}|U^0_\mu |^2d\mu }\\ & \quad +2\alpha \langle \nabla D(y^0),\nu \rangle \int _{\mathbb {R}}|U^0_\mu |^2d\mu -\Xi ^0_t\int _{\mathbb {R}}|U^0_\mu |^2d\mu +\int _{\mathbb {R}}f_1(U^0,0)U^0_\mu d\mu =0. \end{aligned}$$

We put

2.14 $$\begin{aligned} c:= -\dfrac{\displaystyle \int _{-\infty }^{\infty } f_1(\Phi ,0)\Phi _\mu d\mu }{\displaystyle \int _\mathbb {R}|\Phi _\mu |^2d\mu } =\dfrac{\displaystyle \int _0^1 f_1(s,0) ds}{\displaystyle \int _\mathbb {R}|\Phi _\mu |^2d\mu } =\dfrac{\displaystyle \int _0^1 f_1(s,0) ds}{\displaystyle \int _0^1 \sqrt{-2F_0(s)}ds} >0, \end{aligned}$$

where

$$\begin{aligned} F_0(u):=\int \limits _0^uf_0(s)ds. \end{aligned}$$

From (2.14), we have that

$$\begin{aligned} \int \limits _{\mathbb {R}}f_1(U^0,0)U^0_\mu d\mu= & -c\sqrt{D(y^0)}\int \limits _\mathbb {R}|U^0_\mu |^2d\mu . \end{aligned}$$

Using the above equation and

$$\begin{aligned} \Xi ^0_t=-V,\quad \Delta \Xi ^0|_{\Gamma (t)}=(N-1)\kappa ,\quad \int \limits _\mathbb {R}\mu U^0_{\mu \mu }U^0_\mu d\mu =-\dfrac{1}{2}\int \limits _\mathbb {R}|U^0_\mu |^2d\mu , \end{aligned}$$

we obtain

$$\begin{aligned} V=\sqrt{D(y^0)}c-D(y^0)(N-1)\kappa -\left( 2\alpha -\dfrac{1}{2}\right) \langle \nabla D(y^0),\nu \rangle . \end{aligned}$$

Rewriting $y^0$ as *y*, we obtain the singular limit equation:

2.15 $$\begin{aligned} V=\sqrt{D(y)}c-D(y)(N-1)\kappa -\left( 2\alpha -\dfrac{1}{2}\right) \langle \nabla D(y),\nu \rangle ,\quad y\in \Gamma (t). \end{aligned}$$

Summarizing the above arguments, we obtain the following main results.

### Theorem 1

Assume that a solution $\Gamma ^0(t)$ of (2.15) is an $(N-1)$-dimensional smooth hypersurface diffeomorphic to the reference manifold $\bar{\Gamma }$ defined by the boundary of a bounded open set in $\mathbb {R}^N$ for $0\le t\le T$ where $T>0$. The signed distance between *x* and $\Gamma ^0(t)$ is denoted by *d*(*x*, *t*). Let *u* be a solution to (2.7) satisfying

$$\begin{aligned} \left\{ \begin{array}{l} 1-Q_0\varepsilon ^2\le u(x,0)\le 1+Q_0\varepsilon ^2,\quad x\in \{x\in \mathbb {R}^N\ |\ d(x,0)\ge P_0\varepsilon |\log \varepsilon |^2\},\\ -Q_0\varepsilon ^2\le u(x,0)\le 1+Q_0\varepsilon ^2,\quad x\in \{x\in \mathbb {R}^N\ |\ -P_0\varepsilon |\log \varepsilon |^2\le d(x,0)\le P_0\varepsilon |\log \varepsilon |^2\},\\ -Q_0\varepsilon ^2\le u(x,0)\le Q_0\varepsilon ^2,\hspace{0.6cm}\quad x\in \{x\in \mathbb {R}^N\ |\ d(x,0)\le -P_0\varepsilon |\log \varepsilon |^2\} \end{array}\right. \end{aligned}$$

with some positive constants $P_0$ and $Q_0$. Then, there exist positive constants $\varepsilon _0$, $P_1$ and $Q_1$ such that for all $\varepsilon \in (0,\varepsilon _0)$, $0\le t\le T$,

$$\begin{aligned} \left\{ \begin{array}{l} 1-Q_1\varepsilon ^2\le u(x,t)\le 1+Q_1\varepsilon ^2,\quad x\in \{x\in \mathbb {R}^N\ |\ d(x,t)\ge P_1\varepsilon |\log \varepsilon |^2\},\\ -Q_1\varepsilon ^2\le u(x,t)\le 1+Q_1\varepsilon ^2,\quad x\in \{x\in \mathbb {R}^N\ |\ -P_1\varepsilon |\log \varepsilon |^2\le d(x,t)\le P_1\varepsilon |\log \varepsilon |^2\},\\ -Q_1\varepsilon ^2\le u(x,t)\le Q_1\varepsilon ^2,\quad \hspace{0.25cm}\quad x\in \{x\in \mathbb {R}^N\ |\ d(x,t)\le -P_1\varepsilon |\log \varepsilon |^2\}. \end{array}\right. \end{aligned}$$

Define $\Gamma ^{\varepsilon }(t)$ as the level set where $u=1/2$. This theorem implies that $\Gamma ^{\varepsilon }(t)$ remains close to the interface $\Gamma ^{0}(t)$ and that the singular limit problem for (2.7) is given by (2.15). The proof is provided in the appendix.

We often refer to a *wave* as a (moving) interface between two stable states.

### Remark 1

A solution of $u_t=u_{zz}+f(u,\varepsilon )$ that moves at a constant speed without changing its profile is called a *traveling wave solution*. Under the bistable setting (2.8), it is well known that there exists a unique traveling wave solution connecting 1 and 0 at a unique speed, which is denoted by $\phi$. In other words, a traveling wave solution $\phi$ with speed $c(\varepsilon )$ satisfies

$$\begin{aligned} \phi ''(z)+c(\varepsilon )\phi '(z)+f(\phi (z),\varepsilon )=0,\quad \phi (-\infty )=1,\quad \phi (0)=1/2,\quad { \phi (\infty )=0,\quad } \phi '(z)<0. \end{aligned}$$

Then,

$$\begin{aligned} c(\varepsilon )=\dfrac{\displaystyle \int \limits _0^1 f_0(\phi )d \phi +\varepsilon \displaystyle \int \limits _0^1 f_1(\phi ,\varepsilon )d \phi }{\displaystyle \int \limits _{-\infty }^{\infty } (\phi ')^2dz}=\dfrac{\displaystyle \int \limits _0^1 f_1(\phi ,\varepsilon )d \phi }{\displaystyle \int \limits _{-\infty }^{\infty } (\phi ')^2dz }\varepsilon . \end{aligned}$$

By the assumption (2.8), we see that $c(\varepsilon )=c\varepsilon +O(\varepsilon ^2)$ as $\varepsilon \rightarrow 0$ from (2.12). In particular, for the case (2.9), it is well known that

$$\begin{aligned} \Phi (z)=\dfrac{1}{1+e^{z/\sqrt{2}}},\quad c=\sqrt{2} a,\quad c(\varepsilon )=c\varepsilon . \end{aligned}$$

First, we consider the one-dimensional case. The interface position is denoted by *x*(*t*). In this case, (2.15) is rewritten as

2.16 $$\begin{aligned} x'(t)=\sqrt{D(x)}c-\dfrac{1}{2}(4\alpha -1)D'(x). \end{aligned}$$

We define the auxiliary function $\mu$ as follows:

2.17 $$\begin{aligned} \mu (x):=\dfrac{D'(x)}{\sqrt{D(x)}}. \end{aligned}$$

Assume that

2.18 $$\begin{aligned} D'(x)\ge 0,\quad \lim _{|x|\rightarrow \infty }D'(x)=0,\quad \inf _{x\in \mathbb {R}}D(x)>0,\quad \mu ^*:=\sup _{x\in \mathbb {R}}\mu (x)<\infty . \end{aligned}$$

Namely, the diffusivity *D* on the right side is larger than that on the left. Set

$$\begin{aligned} \eta (\mu ):=\dfrac{1}{4}+\dfrac{c}{2\mu }. \end{aligned}$$

Then, (2.16) can be rewritten as follows:

2.19 $$\begin{aligned} x'(t)=2\sqrt{D(x)}\mu (x)(\eta (\mu (x))-\alpha ). \end{aligned}$$

**Fig. 1:**
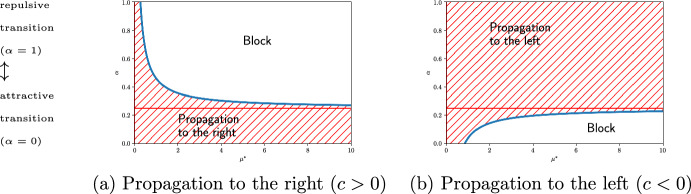
Propagation and blocking in one dimensional case. The horizontal axis represents $$\mu ^*$$, whereas the vertical axis represents $$\alpha$$. Note that the cases $$\alpha =1$$ and $$\alpha =0$$ correspond to repulsive transition and attractive transition, respectively. The (blue) solid curve represents the graph of $$\alpha$$$$\, =\eta (\mu ^*)$$$$\, =(\mu ^*+2c)/(4\mu ^*)$$

If $\alpha \in [0,1/4]$, then region $\{u=1\}$ propagates to the right for any *D*. If $\alpha \in (1/4,\eta (\mu ^*)]$, then region $\{u=1\}$ propagates to the right. If $\alpha \in (\eta (\mu ^*),1]$, then region $\{u=1\}$ is blocked by the equilibrium $x_*$ of $\sqrt{D(x)}c-(4\alpha -1)D'(x)/2=0$ (see Fig. 1). These results can be understood heuristically as follows. The case of $\alpha =0$ corresponds to an attractive transition; thus, when $\alpha$ is small, the interface tends to move rightwards. Conversely, because $\alpha =1$ corresponds to a repulsive transition, the interface moves in the opposite direction as $\alpha$ approaches 1. Hence, it is reasonable that a separatrix $\alpha =\eta (\mu )$ exists. From the above analysis, the separatrix converges to 1/4 as $\mu \rightarrow \infty$. Thus, for any monotone increasing function *D*(*x*) satisfying (2.18), the interfaces propagate to the right $(c>0)$ for $\alpha \in [0,1/4]$ and that ones propagate to the left $(c<0)$ for $\alpha \in [1/4,1]$. Namely, if the transition possibility depends only on the point dividing the departure and the arrival points in the ratio of 3 to 1, interfaces can propagate in both directions independently of *D*. As observed above, a separatrix $\alpha =\eta (\mu )$ lies within the interval $(1/4,\infty )$. From the perspective of bifurcation, as $\alpha$ increases, the saddle node bifurcation occurs at $\alpha =\eta (\mu )$ and new equilibria emerge. One of these emerging equilibria blocks interfaces to the right. When $\alpha \in (1/4,1]$, interfaces propagating to the right may be blocked for certain monotonically increasing functions *D*(*x*). In this case, we will refer to the interface as conditionally blocked. See Fig. 1 for further details.

### Corollary 1

Assume that $N=1$ and *D* satisfies (2.18). When $c>0$ and $\alpha =1/4$, interfaces of (2.16) propagate in both directions as $\varepsilon \rightarrow 0$. When $\alpha \in [0,1/4)$, any interfaces propagate to the right $(c>0)$, and when $\alpha \in (1/4,1]$, the interface propagating to the right $(c>0)$ is conditionally blocked. When $\alpha \in (1/4,1]$, any interfaces propagate to the left $(c<0)$, and when $\alpha \in [0,1/4)$, the interface propagating to the left $(c<0)$ is conditionally blocked.

### Remark 2

Hale and Raugel (1992) studied the reaction-diffusion equation in the thin domain:

2.20 $$\begin{aligned} u_t=\Delta u+g(u),\quad t>0,\ x\in \Omega _\delta \end{aligned}$$

with the Neumann boundary condition on $\partial \Omega _\delta$, where $\delta >0$ and

$$\begin{aligned} \Omega _\delta =\{(x,y)\in \mathbb {R}^{N+1}\ |\ x\in \Omega ,\ 0<y<\delta\, m(x)\}. \end{aligned}$$

Here *m* is a positive smooth function. As $\delta \rightarrow +0$, the dynamics of (2.20) can be approximated by the reduced equation:

2.21 $$\begin{aligned} v_t=\dfrac{1}{m(x)}\nabla (m(x)\nabla v)+g(v),\quad t>0,\ x\in \Omega \end{aligned}$$

with the Neumann boundary condition on $\partial \Omega$.

Applying this argument to (2.7) on the thin domain $\Omega _\delta$ and letting $\delta \rightarrow +0$, we can formally derive

2.22 $$\begin{aligned} v_t&=\dfrac{1}{m(x)}\nabla \Big (m(x)D(x)^{2-2\alpha }\nabla (D(x)^{2\alpha -1}v)\Big ) \\ & \,\quad +\dfrac{1}{\varepsilon ^2}f(v,\varepsilon ),\quad t>0,\ x\in \Omega . \end{aligned}$$

Here, we assume that *D* depends only on $x\in \Omega$ for simplicity. Similarly, we also show that the singular limit problem for (2.22) is given by

2.23 $$\begin{aligned} V&=\sqrt{D(x)} c-\left( 2\alpha -\frac{1}{2}\right) \langle \nu , \nabla D(x)\rangle -\dfrac{D(x)}{m(x)}\langle \nu , \nabla m(x)\rangle \\ & \,\quad -D(x)(N-1) \kappa . \end{aligned}$$

Set $D(x)=1$. Then, (2.23) becomes

2.24 $$\begin{aligned} V=c-\dfrac{1}{m(x)}\langle \nu , \nabla m(x)\rangle -D(x)(N-1) \kappa . \end{aligned}$$

Wave blocking due to the geometry of the domain is also known. When the domain expands abruptly, propagation in a bistable system can be blocked (see Chapuisat and Grenier (2005); Berestycki et al. (2009, 2016)). When *m* increases rapidly in a certain direction $\nu$ such that $\langle \nu , \nabla m(x) \rangle / m(x)$ exceeds *c*, (2.24) also explains that the propagation of the interface is blocked.

If $m=1/D(x)$ and $\alpha =1/2$, then (2.22) is reduced to

$$\begin{aligned} v_t=D(x)\Delta v+\dfrac{1}{\varepsilon ^2}f(v,\varepsilon ),\quad t>0,\ x\in \Omega . \end{aligned}$$

and (2.23) is the same as in the case of an attractive transition.

In contrast to the bistable case, the situation is completely different in the monostable case. The speed of monostable traveling wave solutions has been studied in Weinberger (2002); Berestycki et al. (2008) and the references therein.

This paper is organized as follows. In Sect. 3, we confirm the above results for the singular limit problem (2.15) by using the numerics of the reaction-diffusion equation (2.7) with small $\varepsilon$. As an application of this concept, we consider the Aliev-Panfilov model to explore the mechanism for generating spiral patterns in Sect. 4. In Appendix, we give the proof of Theorem 1 by constructing supersolutions and subsolutions. Sect. 5 provides the concluding remarks and summarizing the main results.

## Numerical simulations

### One-dimensional case

First we consider (2.7) in the one-dimensional interval [0, *L*] under the no-flux boundary condition. The nonlinear function *f* is selected as (2.9), with ${ a_1}=0.3$. For $0<D_{min}<D_{max}$, $\delta >0$, we set

$$\begin{aligned} \zeta (x):= {\left\{ \begin{array}{ll} D_{min}\quad & (x\le -\delta ),\\ \dfrac{D_{max}+D_{min}}{2}+\dfrac{3(D_{max}-D_{min})x}{4\delta }\left( 1-\dfrac{x^{2}}{3\delta ^2}\right) \quad & (|x|< \delta ),\\ D_{max}\quad & (\delta \le x). \end{array}\right. } \end{aligned}$$

Take the diffusion coefficient *D* as

3.1 $$\begin{aligned} D(x):=\zeta (x-L/2). \end{aligned}$$

Thus, *D* monotonically increases in *x*. Note that $$D_{min}= \,$$$$\min _{x\in [0,L]}$$ $$D(x)$$ and $$D_{max}$$ $$\max _{x\in [0,L]}$$ $$D(x)$$ by the definition of *D*. When $$D_{max}$$ $$=1.5, D_{min}=0.5,$$ $$\delta = 0.3,$$ $$L=2$$, we can calculate that $$\mu ^{*}\approx 2.6$$.

**Fig. 2:**
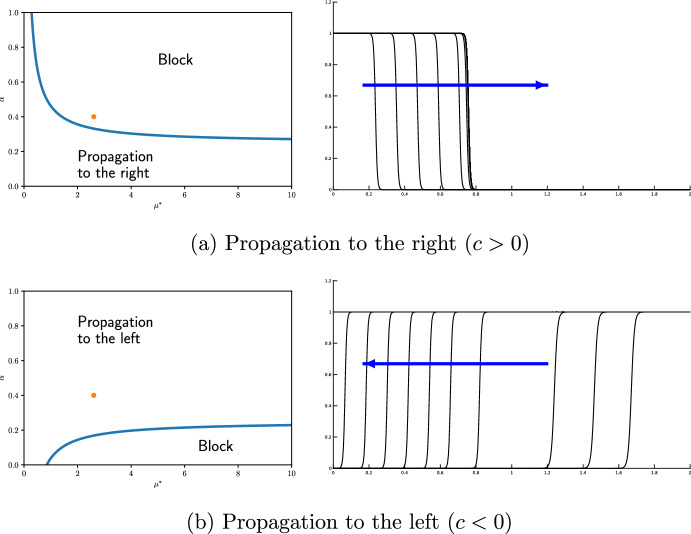
Directional propagation of waves of (2.7) with $$\varepsilon = 0.005,$$$$\ { a_1}=0.3,$$ $$\alpha = 0.4,$$$$\ L=2,$$$$\ D_{max}=1.5,$$$$D_{min}=0.5,$$$$\delta = 0.3$$. The wave propagation, calculated using the parameters indicated by the red dot in the left figure, is shown on the right figure

First we observe the directional propagation. When $\alpha =0.4$, the wave propagates to the left because $\alpha >1/4$. The wave moving to the right is blocked because $\alpha >\eta (\mu ^*)=1/4+\sqrt{2}a/(2\mu ^*)$. These predictions from the singular limit problem are confirmed by numerical calculations of the reaction-diffusion equation (2.7) with a small $\varepsilon$ as shown in Fig. 2. We observe that the cases $\alpha =1/2$ (neutral transition) and $\alpha =1$ (repulsive transition) are identical to those of $\alpha =0.4$.

**Fig. 3:**
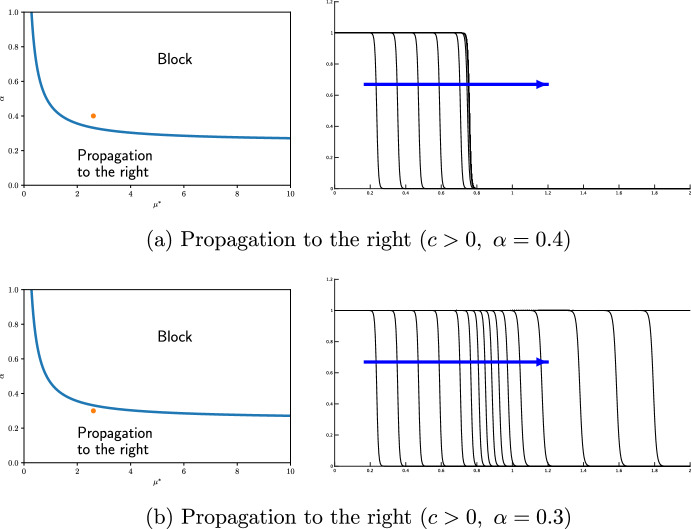
Propagation and blocking near the separatrix $$\mu =\eta (\alpha )$$ under (3.1) ($$\varepsilon =0.005,$$$$\ { a_1}=0.3,$$$$\ L=2,$$$$\ \mu ^{*}\approx 2.6$$). The wave propagation, calculated using the parameters indicated by the red dot in the left figure, is shown in the right figure

Next, we examine the effect of $\alpha$ on propagation. If we take $\alpha =0.4$, then $\alpha >\eta (\mu ^*)$ and the wave can propagate to the right. However, if $\alpha =0.3$, then $\alpha <\eta (\mu ^*)$ and the wave is blocked. Thus, we can confirm that a separator exists between $\alpha =0.3$ and 0.4 as shown in Fig. 3.

We can provide the following summary: Directional propagation is also observed in reaction-diffusion equation (2.7) with small $\varepsilon$. Under repulsive transitions, a wave trends to propagate from an area of larger diffusivity to an area of smaller diffusivity, but not in the opposite direction. On the other hand, under attractive transitions, there is a force that draws towards areas of larger diffusivity, making it difficult for propagation to occur in the reverse direction. These properties are also observed in the reaction-diffusion equation (2.7). It is confirmed that the threshold between propagation and blocking is close to the theoretical value $\alpha =\eta (\mu ^*)$ when $\varepsilon$ is sufficiently small.

### Two-dimensional case

Next, we examine the effect of curvature by studying (2.7) with (2.9) in a two-dimensional rectangle $[0,L]^2$. We extend the definition of $\mu$ in (2.17) to the multi-dimensional case as follows:

$$\begin{aligned} \mu (x,y,\nu ):=\dfrac{\langle \nu ,\nabla D(x,y)\rangle }{ \sqrt{D(x,y)}}, \end{aligned}$$

where $\nu$ denotes the outer normal vector at $(x,y)\in \Gamma (t)$. The interface equation (2.15) can be rewritten as follows:

3.2 $$\begin{aligned} V=\sqrt{D(x,y)} \left( c-\frac{4\alpha -1}{2}\mu (x,y,\nu )-(N-1)\sqrt{D(x,y)} \kappa \right) , \end{aligned}$$

where $c=\sqrt{2}\,a$. If *D* and the initial function *u*(*x*, *y*, 0) depend only on *y*, then the solution also depends on *y* and the curvature vanishes. This implies that it becomes a planar wave and the situation is the same as in the one-dimensional case discussed in the previous subsection.

**Fig. 4:**
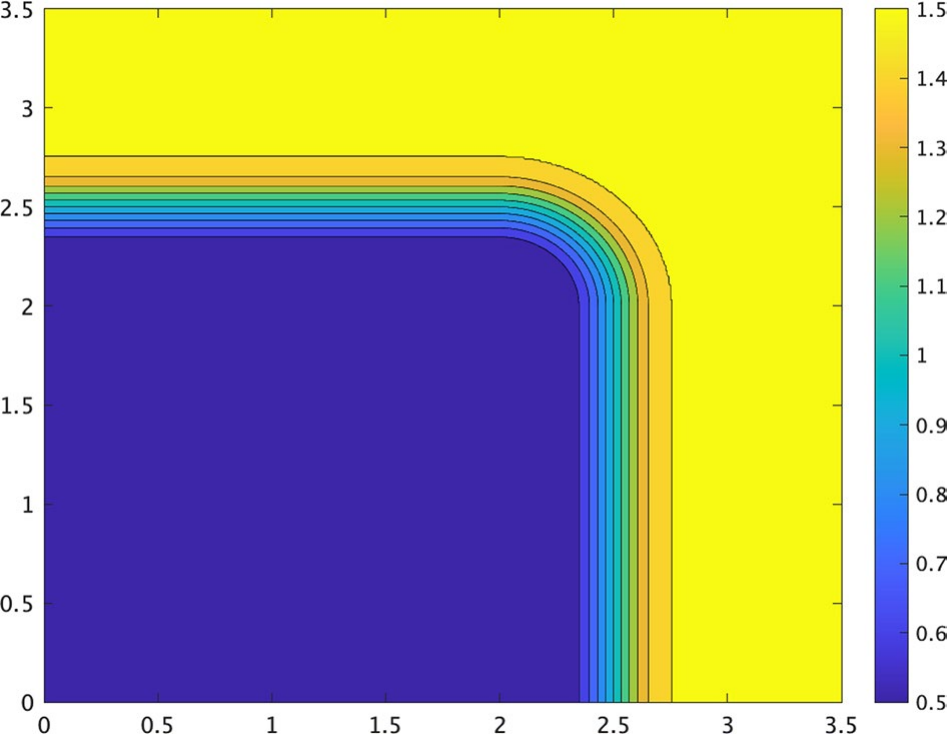
Contours of *D*(*x*, *y*) given by (3.3) with $$L_{0}=2,$$$$\ R=0.5,\ L=3.5,$$$$\ D_{max}=1.5,$$$$\ D_{min}=0.5,\ \delta =0.25$$

To examine how the propagation changes when the contours of the diffusion coefficient *D* are curved, we need to introduce *D*(*x*, *y*). We set the parameters $D_{max},\ D_{min}$ and $\delta$ as in the previous section. If $\alpha =0$, then a one-dimensional wave, as well as a planar wave, cannot propagate from an area of larger diffusivity to one of smaller diffusivity owing to the attractive transition probability as shown in Fig. 2. Now, to curve its contours, we take the diffusion coefficient *D* as follows:

3.3 $$\begin{aligned} D(x,y):= {\left\{ \begin{array}{ll} \zeta (y-L_0-R)\quad & (x\le L_0),\\ \zeta (x-L_0-R)\quad & (y\le L_0),\\ \zeta (\sqrt{(x-L_0)^2+(y-L_0)^2}-R)\quad & (\hbox {otherwise}), \end{array}\right. } \end{aligned}$$

where $L_0$ and *R* are positive constants that satisfy $L_0+R+\delta <L$. The level set of *D*(*x*, *y*) has a corner, as shown in Fig. 4.

**Fig. 5:**
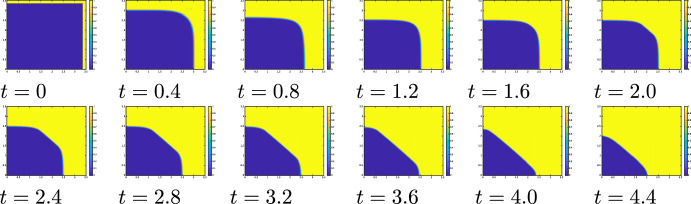
Numerical solution *u*(*x*, *t*) of (2.7) with (2.9) and (3.3) propagates by curvature effect even though it cannot propagate in one-dimensional case ($\varepsilon = 0.01,\ { a_1}=1,\ \alpha = 0$)

Recall that, if $D(x,y)=\zeta (y-L_0-R)$, then the downward planar wave is blocked. When the contours of the diffusion coefficient *D* are curved, the numerical simulation of (3.2) with (3.3) and $\alpha =0$ is shown in Fig. 5. The situation changes owing to the effect of the curvature. In regions where the contours of *D* are flat, waves are temporarily blocked. However, in the regions where the contours are curved, the wave manages to penetrate areas of lower diffusivity owing to the curvature effect, leading to an overall propagation (Fig. 5).

## Application to the Aliev–Panfilov model

The Aliev-Panfilov model is one of mathematical models to describe the electrical activity of cardiac tissue. It demonstrates the propagation of electrical impulses in the heart and is essential for understanding cardiac arrhythmias. By introducing variable diffusion into the Aliev-Panfilov model, we have

4.1 $$\begin{aligned} \left\{ \begin{array}{rcl} \dfrac{\partial u}{\partial t} & =& {{\mathcal {L}}}_{D,\alpha }u-ku(u-a)(u-1)-uv,\\ \dfrac{\partial v }{\partial t}& =& \Big (\varepsilon _0+\dfrac{\mu _1v}{u+\mu _2}\Big )\Big ({-v-ku(u-b-1)}\Big ), \end{array}\right. \end{aligned}$$

where *u* and *v* represent the transmembrane potential and the recovery variable respectively. Here $\mu _1,\mu _2, k, a, b, \varepsilon _0$ are non-negative constants. Because the comparison theorem does not hold for the Aliev-Panfilov model, the results in the previous section cannot be used directly. Therefore, by inferring the front dynamics of the Aliev-Panfilov model from the results of the singular limit problem of the Allen-Cahn-Nagumo equation, we will explain the numerical calculation results. In this section, we refer to the region where the variable *u* takes relatively large values as the wave. This corresponds to the excited state in the context of action potential propagation.

Now, we take

**Fig. 6:**
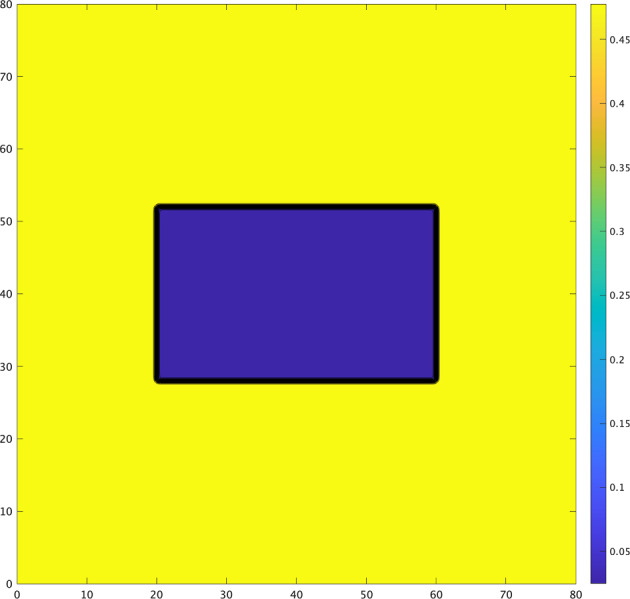
The contour of *D*(*x*, *y*) given by (4.2) where $| \, y_{1}-y_{2} \,| =24$ $| \, y_{1}-y_{2} \,| =24$, $\ D_{min} = 0.025$ $\ D_{min} = 0.025$ and $\delta =1/3.467$

4.2 $$\begin{aligned} D(x,y)= & D_{max}-\dfrac{D_{max}-D_{min}}{ 4}\Big (1+\tanh \dfrac{x-x_{1}}{\delta }\Big )\Big (1+\tanh \dfrac{x_{2}-x}{\delta }\Big ) \nonumber \\ & \hspace{10pt}\times \Big (1+\tanh \dfrac{y-y_{1}}{\delta }\Big )\Big (1+\tanh \dfrac{y_{2}-y}{\delta }\Big ), \end{aligned}$$

where $D_{max}=0.5,\ D_{min} = 0.025$ and $\delta =1/3.467$, whose level sets are shown in Fig. 6. We see that there is a central rectangular area with lower diffusivity inside the domain.

**Fig. 7:**
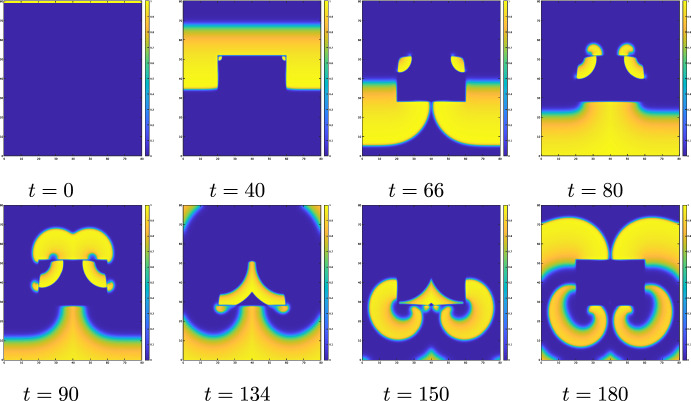
Dynamics of the numerical solution *u*(*x*, *y*, *t*) of (4.1) with (4.2) when $$\alpha = 0$$$$k=8,\ \varepsilon _0=0.01,$$$$\mu _1=0.15,$$$$\ \mu _2=0.3,\ a=b=0.1$$

The numerical results of (4.1) with (4.2) and $\alpha =0$ are shown in Fig. 7. The wave propagates downward $(t=0)$. When $\alpha =0$, the wave cannot propagate from area of larger diffusivity to areas of smaller diffusivity. Therefore, the wave cannot enter the central rectangular area of lower diffusivity from any of its sides. However, due to the influence of curvature, the wave can enter at the corners, allowing the wave to propagate further ($t=40$). Note that due to the differences in diffusivity, the width of the pulses and their speeds also vary. The wave can propagate from areas of the lower diffusivity to that of the larger diffusivity, but it cannot propagate if the inhibitor remains present $(t=66)$. After the inhibitor dissipates, the wave begins to propagate into areas of larger diffusivity $(t=80)$. The wave that has spread into these areas eventually reaches the corners of regions with lower diffusivity $(t=90)$. Due to the effect of the inhibitor, the wave can no longer enter from the corners. The waves on the top and sides of the rectangle at $t=90$ merge and propagate downward. Due to differences in wave speeds inside and outside the rectangle, a new wave emerges near the previous wave $(t=134)$. This new wave forms a spiral due to the influence of the inhibitor $(t=134,150)$, and the spiral wave remains stable $(t=180)$. Therefore, when $\alpha <1/4$, spiral waves may be generated by the same process because the direction of propagation is same.

Next, we consider the case $\alpha >1/4$. The direction of propagation is reversed. A wave can propagate from an area of higher diffusivity to an area of lower diffusivity and it propagates faster in an area of higher diffusivity than that of lower diffusivity, but it cannot propagate from the area of lower diffusivity to that of higher diffusivity. Therefore, it is rather delicate to construct an example that creates target patterns as described above. We take

4.3 $$\begin{aligned} & {D(x,y)}\nonumber \\&=  \, D_{min}+\dfrac{D_{max}-D_{min}}{4}\Big (1+\tanh \dfrac{x-x_{1}}{\delta }\tanh \dfrac{x_{2}-x}{\delta }\Big )\Big (1+\tanh \dfrac{y-y_{1}}{\delta }\tanh \dfrac{y_{2}-y}{\delta }\Big )\nonumber \\ & \quad +\dfrac{D_{max}-D_{min}}{4}\Big (1+\tanh \dfrac{x-x_{1}}{\delta }\tanh \dfrac{x_{2}-x}{\delta }\Big ) \Big (1+\tanh \dfrac{y-y_{4}}{\delta }\tanh \dfrac{y_{3}-y}{\delta }\Big ), \end{aligned}$$

where $$D_{max}=3, D_{min} = 0.04$$ and $$\delta = 1/3.38$$.

**Fig. 8:**
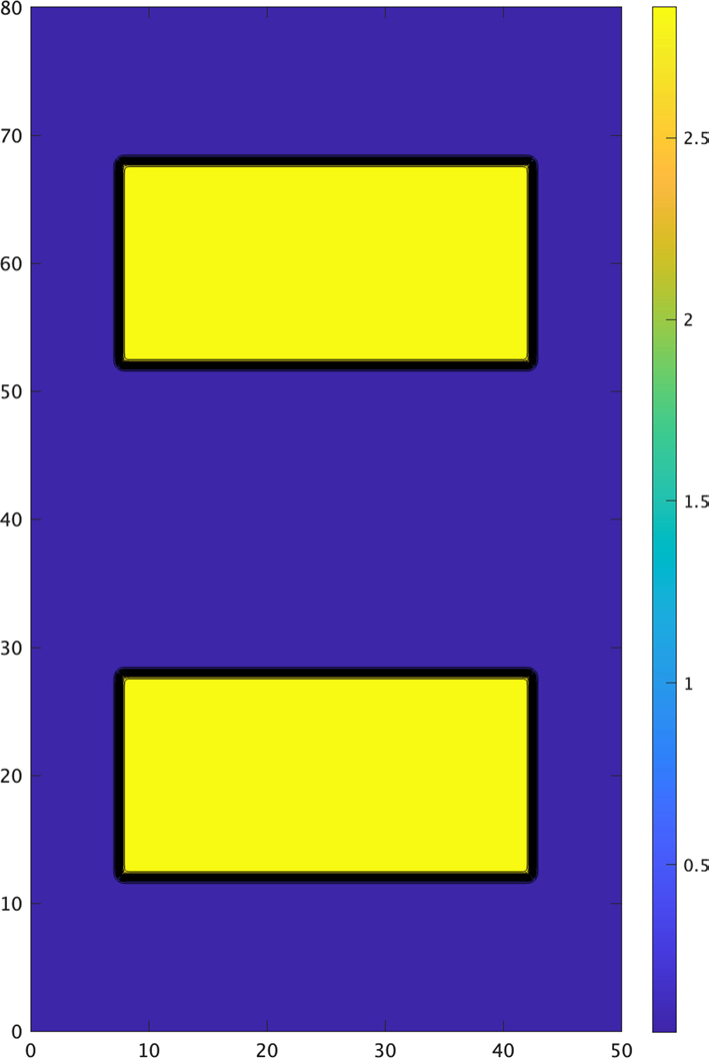
The contour of *D*(*x*, *y*) given by (4.3) where $$\left| \, x_{1}-x_{2} \,\right| =35, \left| \, y_{1}-y_{2} \,\right| =16, \left| \, y_{3}-y_{4} \,\right|$$$$=16, \left| \, y_{2}-y_{3} \,\right| =24$$, $$D_{max}=3, D_{min} = 0.04$$, and $$\delta = 1/3.38$$

**Fig. 9:**
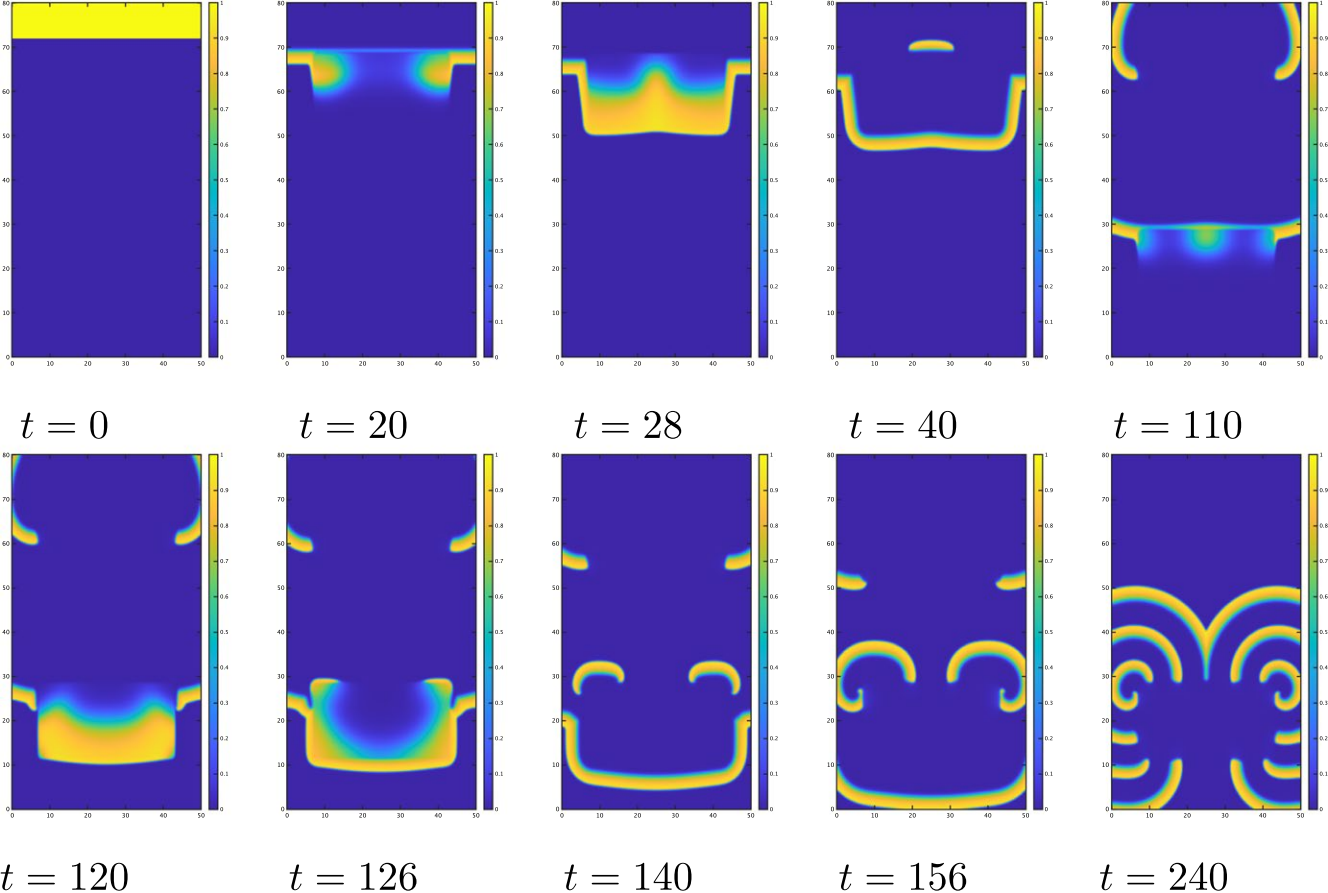
Dynamics of the numerical solution *u*(*x*, *y*, *t*) of (4.1) with (4.3) when $$\alpha =0.5,$$$$\ k=8,\ \varepsilon _0=0.1,$$$$\mu _1=0.11,\ \mu _2=0.3,$$$$\ a=b=0.1$$

The level sets of *D* have two rectangular peaks, as shown in Fig. 8. The numerical results of (4.1) with (4.3) and $\alpha =0.5$ are shown in Fig. 9. The wave propagates downward and hits to the central rectangular area of higher diffusivity. When $\alpha =0.5$, the wave cannot propagate from an area of lower diffusivity to an area of lower diffusivity. As a result, the wave cannot enter the central rectangular area of higher diffusivity from any of its sides. However, due to the influence of curvature, it can enter at the corners $(t=20)$. A wave can propagate faster in the central rectangular area of higher diffusivity than the other area and then it can propagate to an area of lower diffusivity. It can also propagate to the upper side of the central rectangular area after the inhibitor dissipates $(t=28)$. The wave that has spread into the upper area of lower diffusivity continues to propagate, resulting in the existence of two waves $(t=40)$. The lower wave reaches the lower rectangular region at $t=110$. Upon hitting the upper side of this lower rectangular region, the wave front is not flat but concave. Due to the curvature effect, the wave succeeds to enter at the center of the upper side at $t=110$. The wave that enters the lower rectangle spreads toward both lower sides and then moves upward to the upper side $(t=120,126)$. The newly generated two waves propagate $(t=140)$, resulting in the existence of five waves. These new waves form a spiral due to the influence of the inhibitor $(t=156)$, which remains stable at $t=240$. Although spiral waves are less likely to be generated for $\alpha >1/4$ compared to $\alpha <1/4$, a diffusion coefficient with two peaks can still generate spiral waves.

### Remark 3

If the value of $\delta$ in (4.2) varies on each side, the spiral wave can be easily generated. For example, let

4.4 $$\begin{aligned} D(x,y)= & D_{min}+\dfrac{D_{max}-D_{min}}{4}\Big (1+\tanh \dfrac{x-x_{1}}{\delta _1}\tanh \dfrac{x_{2}-x}{\delta _2}\Big )\nonumber \\ & \quad \times \Big (1+\tanh \dfrac{y-y_{1}}{\delta _3}\tanh \dfrac{y_{2}-y}{\delta _4}\Big ). \end{aligned}$$

**Fig. 10:**
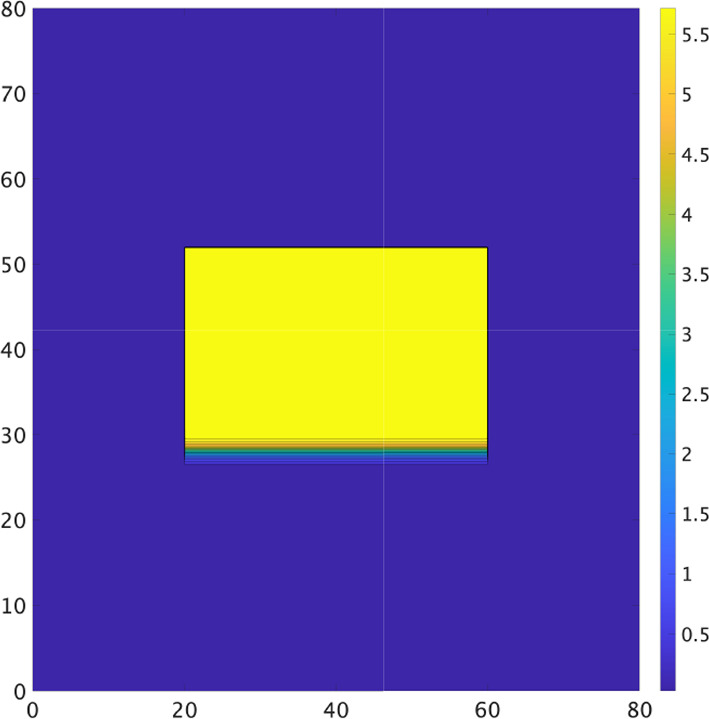
The contour of *D*(*x*, *y*) given by (4.4) where $\left| \, x_{1}-x_{2} \,\right| =40, \left| \, y_{1}-y_{2} \,\right| =30$, $D_{max}=6, D_{min} = 0.025$, and $\delta _1=\delta _2=\delta _4 = 40$, $\delta _3=1$

**Fig. 11:**
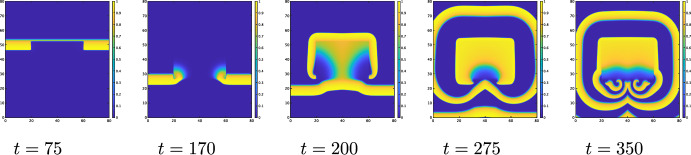
Dynamics of the numerical solution *u*(*x*, *y*, *t*) of (4.1) with (4.3) when $$\alpha =0.5,$$$$\ k=8,\ \varepsilon _0=0.1,$$$$\ \mu _1=0.15,\ \mu _2=0.3,$$$$\ a=b=0.1$$

The contours plot of *D* is shown in Fig. 10. In the square region with higher diffusion, wave propagation is blocked from the outside along the top and lateral edges, while propagation from the inside is allowed. Additionally, the diffusion coefficient along the bottom edge is set to allow propagation from the outside into the region. Under these conditions, spiral waves are observed to emerge. The corresponding numerical result is shown in Fig. 11.

## Concluding remarks

In this paper, we introduced the variable diffusion equation (2.7), following the argument by Okubo and Levin (2001), where the transition probability depends on a point between the departure and arrival points. As a result, this equation serves as an interpolation between repulsive and attractive transitions. It can also be derived from other perspectives, such as those presented in Alfaro et al. (2022); Wereide (1914). In our argument in Sect. 2 (or Okubo and Levin (2001)), the jump lengths are assumed to have the same variance over equal time intervals. On the other hand, Visser (2008) derived the following equation by assuming that the jump length depends on a point between the departure and arrival points:

5.1 $$\begin{aligned} u_t=(1-\beta )(D_x(x)u)_x+ (D(x)u_x)_x. \end{aligned}$$

By setting $\beta = 2(1 - \alpha )$, this equation coincides with (2.2). In this way, equation (2.7) arises from various different problems.

Next, we investigated the singular limit of (2.7) as $\varepsilon \rightarrow +0$. In this limit, the equation reduces to (2.15). Corollary 1 describes the dynamics of (2.15) in the one-dimensional case. We now reinterpret Corollary 1 from the perspective of mathematical biology. In the case of attractive transition, biological species tend to propagate from regions with lower diffusion coefficients toward regions with higher diffusion coefficients. Conversely, under repulsive transition, propagation typically proceeds from regions of higher diffusion to those of lower diffusion. Although propagation in the opposite direction is generally more difficult, it is not entirely precluded. At first glance, this may suggest that the magnitude of the diffusion coefficient plays a decisive role; however, this is not necessarily the case. In the repulsive case, propagation can still occur if $\mu ^*$ is sufficiently small. This observation suggests that the magnitude of the diffusion coefficient alone does not solely determine the propagation behavior; rather, the spatial variation of the diffusion are also essential factors. Let $D_{min}$ and $D_{max}$ denote the minimum and maximum values of the diffusion coefficient *D*(*x*) on the interval [0, *L*], respectively. In this case, $D_{min}=D(0)$ and $D_{max}=D(L)$. Consider the larger domain $[0,L']$ with $L'>L$ and define the rescaled diffusion coefficient on this extended domain by $D(x;L')=D(Lx/L')$ for $x\in [0,L']$. Then, the rescaled diffusion coefficient $D(x;L')$ retains the same range of values as *D*(*x*), while its spatial variation becomes more gradual as $L'$ increases. As a result, $\mu ^*$ decreases. Therefore, even when $D_{min}$ and $D_{max}$ are fixed, wave propagation may still occur if the domain is sufficiently wide, that is, the gradient of *D*(*x*) becomes gentle. In contrast, if the domain is narrow, i.e., the gradient of *D*(*x*) becomes steep, $\mu ^*$ becomes large, which may prevent propagation. In other words, if the diffusion coefficient increases gradually, species with repulsive transition can propagate; however, if the diffusion coefficient increases rapidly, propagation becomes impossible. Similar phenomena are observed in the attractive transition case when $c<0$. If the diffusion coefficient decreases gradually, species with attractive transition can invade; however, if the diffusion coefficient increases rapidly, propagation becomes impossible.

In the two-dimensional case, propagation may still occur due to the effect of curvature, even in situations where it would be blocked in one dimension. This phenomenon is also confirmed numerically in Sect. 3.2. We further applied the approach to the Alieve–Panfilov model, which admits traveling pulse solutions consisting of the front and the back. Since the dynamics of the front can be predicted from the motion of the interface in the Allen–Cahn–Nagumo equation, we attempted to predict the solution dynamics of the Aliev-Panfilov model with the heterogeneous diffusion coefficients. Based on this insight, we constructed the examples of the heterogeneous diffusion coefficients to create the spiral patterns in Sect. 4.

## Data Availability

This manuscript has no associated data.

## Appendix

The heuristic derivation of (2.15) was stated in Sect. 2. The rigorous proof of Theorem 1 is based on the maximum principle (see also Matano (1979)) and construction of supersolutions and subsolutions.

### Proof of Theorem 1

Since the proof is based on Theorem 2.1 of Ei et al. (1997) (see also Theorem 3 of Chen (1992)), we only give an essential part of proof here. Set

$$\begin{aligned} {{\mathcal {F}}}[u]:= & u_t-{{\mathcal {L}}}_{D,\alpha } u-\dfrac{1}{\varepsilon ^2}f(u,\varepsilon ), \end{aligned}$$

where ${{\mathcal {L}}}_{D,\alpha }$ is defined in (2.4). For the readers’ convenience, we reiterate the notation in Ei et al. (1997). Let $\Gamma ^+(t)$ be a one-parameter family of hypersurfaces diffeomorphic to $\bar{\Gamma }$ which is a supersolution of the interface equation (2.15). Namely, $\Gamma ^+(t)$ satisfies

A.1 $$\begin{aligned} V\ge & \sqrt{D(y)}c-\dfrac{1}{2}(4\alpha -1)\langle \nu ,\nabla D(y)\rangle -D(y)(N-1)\kappa +R\varepsilon (\log \varepsilon )^2 \end{aligned}$$

with some positive constant *R* (see Lemma 4.2 of Ei et al. (1997)).

To construct a supersolution of (2.7), we introduce another signed distance function from *x* to $\Gamma ^+(t)$, which is measured with the metric

$$\begin{aligned} ds^2=\dfrac{1}{D(\cdot )}(dx_1^2+\cdots +dx_N^2). \end{aligned}$$

It is denoted by $\eta (x;\Gamma ^+(t))$. Then $|\eta |$ is the infimum of the length of geodesics from *x* to $\Gamma ^+(t)$. Let $y^+\in \Gamma ^+(t)$ be an end point of the geodesic from *x* to $\Gamma ^+(t)$. We have the following relation between $\eta$ and $\xi =|{ y^+}-x|$:

$$\begin{aligned} \eta =\dfrac{\xi }{\sqrt{D(x)}}-\dfrac{\xi ^2}{2}\left\langle \nu ,\nabla \dfrac{1}{\sqrt{D(x)}}\right\rangle +O(\xi ^3) \end{aligned}$$

as $\xi \rightarrow 0$. The signed distance function and $\eta$ can be constructed near $\Gamma ^\varepsilon (t)$. Therefore, we need to modify the nonlinear function $f(u,\varepsilon )$ to $f(u,\varepsilon )+R_0\varepsilon ^2$. We choose a positive constant $R_0$ satisfying

A.2 $$\begin{aligned} R_0\ge 3\sup _{x\in \mathbb {R}^N}|\Delta D(x)|. \end{aligned}$$

Let $b_j(\varepsilon )$ $(j=1,2,3)$ be the zeros of the perturbed nonlinear term, where $b_1(\varepsilon )<b_2(\varepsilon )<b_3(\varepsilon )$ and $b_1(\varepsilon )=O(\varepsilon ^2),\ b_3(\varepsilon )=1+O(\varepsilon ^2)$. Then, there is a positive constant $Q_0$ depending on $R_0$ such that

$$\begin{aligned} b_1(\varepsilon )>2Q_0\varepsilon ^2,\quad b_3(\varepsilon )>1+2Q_0\varepsilon ^2. \end{aligned}$$

We also use the traveling wave solution $\Psi$ connecting $b_1(\varepsilon )$ and $b_3(\varepsilon )$ instead of $\Phi$ in (2.12). By denoting the speed of the traveling wave solution $\Psi$ by ${\tilde{c}}(\varepsilon )$, $\Psi$ satisfies

A.3 $$\begin{aligned} &\Psi ''(z)+{\tilde{c}}(\varepsilon )\Psi '(z)+{ f(\Psi (z),\varepsilon )}+R_0\varepsilon ^2=0,\\ &\Psi (-\infty )=b_3(\varepsilon ),\ \Psi (\infty )=b_1(\varepsilon ),\quad \end{aligned}$$

where ${\tilde{c}}(\varepsilon )=c\varepsilon +O(\varepsilon ^2)$ (see also Remark 1). For small $\varepsilon >0$, there are positive constants *C* and *p* such that

A.4 $$\begin{aligned} \Psi '(z)<0, \quad |\Psi ''(z)|\le C\Psi '(z)\quad \hbox { for }z\in \mathbb {R}\end{aligned}$$

and

A.5 $$\begin{aligned} \left\{ \begin{array}{l} b_3(\varepsilon )-\varepsilon ^{3}<\Psi (z)<b_3(\varepsilon ),\quad |\Psi '(z)|<\varepsilon ^{3}\quad \hbox { for }z<-p|\log \varepsilon |,\\ b_1(\varepsilon )<\Psi (z)<b_1(\varepsilon )+\varepsilon ^{3},\quad |\Psi '(z)|<\varepsilon ^{3}\quad \hbox { for }z>p|\log \varepsilon | \end{array}\right. \end{aligned}$$

(see Lemma 4.1 of Ei et al. (1997)). To construct a supersolution, we put

$$\begin{aligned} z(x,t):=p|\log \varepsilon | h\left( \dfrac{\eta (x;\Gamma ^+(t))}{p\varepsilon |\log \varepsilon |}-3\right) \end{aligned}$$

and define

$$\begin{aligned} U^+(x,t):=\Psi (z(x,t)), \end{aligned}$$

where *h* is a smooth function satisfying

$$\begin{aligned} \begin{array}{ll} h(s)=-2\quad & \hbox { for }s\le -3,\\ h(s)=z\quad & \hbox { for }s\in [-1,1],\\ h(s)=2\quad & \hbox { for }s\ge 3 \end{array} \end{aligned}$$

and $$0\le h'(s)\le 1$$, $$|h''(s)|\le 1$$ for $$s\in \mathbb {R}$$.

First, we consider the case where $2p\varepsilon |\log \varepsilon |\le \eta \le 4p\varepsilon |\log \varepsilon |$. In this case, $z(x,t)=\varepsilon ^{-1}\eta -3p|\log \varepsilon |\in [-p|\log \varepsilon |,p|\log \varepsilon |]$. For simplicity of notation we use $z=z(x,t)$. Then, we have

$$\begin{aligned} {{\mathcal {F}}}[U^+]= & z_t\Psi '(z) -D(x)\nabla ( \Psi '(z)\nabla z)\\ & \quad -2\alpha \langle \nabla D(x), \Psi '(z)\nabla z\rangle -(2\alpha -1)(\Psi (z))\Delta D(x)-\dfrac{1}{\varepsilon ^2}f(\Psi (z),\varepsilon )\\= & z_t\Psi '(z) -D(x) \Psi ''(z)|\nabla z|^2-D(x)\Psi '(z)\Delta z\\ & \quad -2\alpha \langle \nabla D(x), \nabla z\rangle \Psi '(z)-(2\alpha -1)\Psi (z)\Delta D(x)-\dfrac{1}{\varepsilon ^2}f(\Psi (z),\varepsilon ). \end{aligned}$$

Note that

$$\begin{aligned} \nabla \xi =\nu ,\quad \xi _t=-V,\quad \Delta \xi =(N-1)\kappa +O(\xi ) \end{aligned}$$

as $\xi \rightarrow 0$. As in the proof of Lemma 4.2 of Ei et al. (1997), we have

$$\begin{aligned} \nabla \eta= & \dfrac{\nabla \xi }{\sqrt{D(x)}}+\xi \nabla \left( \dfrac{1}{\sqrt{D(x)}}\right) -\xi \left\langle \nu ,\nabla \dfrac{1}{\sqrt{D(x)}}\right\rangle \nu +O(\xi ^2),\\ |\nabla \eta |^2= & \dfrac{1}{D(x)}+O(\xi ^2),\\ \Delta \eta= & \dfrac{\Delta \xi }{\sqrt{D(x)}} +2\left\langle \nabla \xi ,\nabla \left( \dfrac{1}{\sqrt{D(x)}}\right) \right\rangle +\xi \Delta \left( \dfrac{1}{\sqrt{D(x)}}\right) -\langle \nabla \xi ,\nu \rangle \left\langle \nu ,\nabla \dfrac{1}{\sqrt{D(x)}}\right\rangle +O(\xi ^2)\\= & \dfrac{(N-1)\kappa }{\sqrt{D(y)}}+\left\langle \nu ,\nabla \left( \dfrac{1}{\sqrt{D(y)}}\right) \right\rangle +O(\xi ),\\ \eta _t= & -\left( \dfrac{1}{\sqrt{D(y)}}+O(\xi )\right) V+O(\xi ^2). \end{aligned}$$

Using $z_t=\eta _t/\varepsilon ,\ \nabla z=\nabla \eta /\varepsilon ,\ \Delta z=\Delta \eta /\varepsilon$, we obtain

$$\begin{aligned} {{\mathcal {F}}}[U^+]= & -\dfrac{V}{\varepsilon \sqrt{D(y)}}\Psi '(z) -\dfrac{1}{\varepsilon ^2}\Psi ''(z) -\dfrac{N-1}{\varepsilon }\sqrt{D(y)}\Psi '(z)\kappa \\ & \quad -\dfrac{1}{\varepsilon }D(y)\Psi '(z)\left\langle \nu ,\nabla \left( \dfrac{1}{\sqrt{D(y)}}\right) \right\rangle -\dfrac{2\alpha }{\varepsilon } \left\langle \nabla D(y),\dfrac{\nu }{\sqrt{D(y)}}\right\rangle \Psi '(z)\\ & \quad -(2\alpha -1)\Psi (z)\Delta D(x)-\dfrac{1}{\varepsilon ^2}f(\Psi (z),\varepsilon ) +O\left( \dfrac{\xi }{\varepsilon }\right) |\Psi '(z)|\\= & -\dfrac{V}{\varepsilon \sqrt{D(y)}}\Psi '(z) -\dfrac{N-1}{\varepsilon }\sqrt{D(y)}\Psi '(z)\kappa -\dfrac{4\alpha -1}{2\sqrt{D(y)}\,\varepsilon } \left\langle \nabla D(y),\nu \right\rangle \Psi '(z)\\ & \quad -(2\alpha -1)\Psi (z)\Delta D(x)+\dfrac{{\tilde{c}}(\varepsilon )}{\varepsilon ^2}\Psi '(z)+R_0+O\left( \dfrac{\xi }{\varepsilon }\right) |\Psi '(z)|. \end{aligned}$$

Here we used (A.3). By (A.2), $|(2\alpha -1)\Psi (z)\Delta D(x)|\le 2R_0/3$. Hence, we obtain

$$\begin{aligned} {{\mathcal {F}}}[U^+]\ge & \dfrac{R_0}{3}-\dfrac{\Psi '(z)}{\varepsilon \sqrt{D(y)}}\Big (V+(N-1)D(y)\kappa -c\sqrt{D(y)}+\dfrac{4\alpha -1}{2} \left\langle \nabla D(y),\nu \right\rangle +O(\xi )\Big )\\\ge & \dfrac{R_0}{3}+\dfrac{|\Psi '(z)|}{\varepsilon \sqrt{D(y)}}\Big (R\varepsilon |\log \varepsilon |^2+O(\varepsilon |\log \varepsilon |)\Big )>0 \end{aligned}$$

for small $\varepsilon >0$ and $2p\varepsilon |\log \varepsilon |\le \eta \le 4p\varepsilon |\log \varepsilon |$.

Next, consider the case where $$\eta <2p$$$$\varepsilon$$$$\log \varepsilon |$$ or $$\eta >4p$$$$\varepsilon$$$$|\log \varepsilon |$$. In this case, (A.5) holds. Then, $$b_3(\varepsilon )$$ $$-\varepsilon ^3\le U^+$$ $$\le b_3(\varepsilon )$$ or $$b_1(\varepsilon )$$$$\le U^+\le b_1(\varepsilon )+\varepsilon ^3$$$$b_1(\varepsilon )\le U^+$$$$\le b_1(\varepsilon )+\varepsilon ^3$$, respectively. There is a positive constant *C* such that $$f(U^+,\varepsilon )+R_0\varepsilon ^2\le C\varepsilon ^3$$ for sufficiently small $$\varepsilon >0$$. Moreover, we have $$|\Psi '(z)|<\varepsilon ^3$$ and $$|\Psi ''(z)|\le C\varepsilon ^3$$. For $$0\le \eta <2p\varepsilon |\log \varepsilon |$$ or $$4p\varepsilon |\log \varepsilon |<\eta \le 6p\varepsilon |\log \varepsilon |$$, we have $$\nabla z=h'\nabla \eta /\varepsilon$$, $$\Delta z=h''|\nabla \eta |^2/(p\varepsilon ^2|\log \varepsilon |)+h'\Delta \eta /\varepsilon$$. Using (A.2) and (A.5), we get

$$\begin{aligned} {{\mathcal {F}}}[U^+]= & z_t\Psi '(z) -D(x) \Psi ''(z)|\nabla z|^2-D(x)\Psi '(z)\Delta z\\ & \quad -2\alpha \langle \nabla D(x), \nabla z\rangle \Psi '(z)-(2\alpha -1)\Psi (z)\Delta D(x)-\dfrac{1}{\varepsilon ^2}f(\Psi (z),\varepsilon )\\= & \dfrac{\eta _t}{\varepsilon }h'\Psi '(z) -D(x) \Psi ''(z)\dfrac{|h'|^2|\nabla \eta |^2}{\varepsilon ^2}-D(x)\Psi '(z)\left( \dfrac{h''|\nabla \eta |^2}{p\varepsilon ^2|\log \varepsilon |}+\dfrac{h'\Delta \eta }{\varepsilon }\right) \\ & \quad -\dfrac{2\alpha }{\varepsilon } \langle \nabla D(x), h'\nabla \eta \rangle \Psi '(z)-(2\alpha -1)\Psi (z)\Delta D(x)-\dfrac{1}{\varepsilon ^2}f(\Psi (z),\varepsilon )\\\ge & -C\varepsilon -\dfrac{C\varepsilon }{|\log \varepsilon |}-\dfrac{R_0}{3}\max \{b_3(\varepsilon ),b_1(\varepsilon )+\varepsilon ^3\}+R_0-C\varepsilon >0 \end{aligned}$$

with some positive constant *C*. For $\eta <0$ or $\eta >6p\varepsilon |\log \varepsilon |$, we have $z=-2p|\log \varepsilon |$ or $z=2p|\log \varepsilon |$ respectively. Since $z_t=0,\ \nabla z=0,\ \Delta z=0$, we can obtain ${{\mathcal {F}}}[U^+]>0$ similarly. The above arguments imply that $U^+$ is a supersolution satisfying $u\le U^+$. We can construct a subsolution similarly. Therefore, the proof of Theorem 1 is completed. $\square$
